# Experimental Study of the Mechanical and Microstructure Characteristics of Coal Gangue Road Stabilization Materials Based on Alkali Slag Cementation

**DOI:** 10.3390/ma14133601

**Published:** 2021-06-28

**Authors:** Changbai Wang, Junxin Yang, Shuzhan Xu

**Affiliations:** School of Civil Engineering and Architecture, Anhui University of Science and Technology, 70 Xueyuan S Rd, Tianjia’an District, Huainan 232001, China; 15856995257@163.com (J.Y.); shuzhanww@163.com (S.X.)

**Keywords:** alkali-activated slag, coal gangue, road stabilization material, mechanical properties, microstructure

## Abstract

To accelerate the resource utilization of coal gangue and meet the strategic requirements of carbon neutralization, alkali-activated, slag-cemented coal gangue is applied in the preparation of solid waste-based road stabilization materials. Here, the cementation characteristics and microstructure characteristics of alkali-activated, slag-cemented coal gangue road stabilization materials are studied using the alkali equivalent and coal gangue aggregate ratio as experimental variables. The results show that with the increase in alkali equivalent from 1% to 7%, the unconfined compressive strength of the alkali-activated coal gangue road stabilization material initially increases and then decreases, with 3% being the optimal group in terms of stabilization, the aggregate ratio of coal gangue increases from 70% to 85%, and the 7-day unconfined compressive strength of the stabilized material decreases approximately linearly from 8.16 to 1.68 MPa. At the same time, the porosity gradually increases but still meets the requirements of the specification. With the increase in hydration time, a large number of hydration products are formed in the alkali slag cementation system, and they are closely attached to the surface of and interweave with the coal gangue to fill the pores, resulting in the alkali slag slurry and coal gangue being brought closer together.

## 1. Introduction

Given its huge output and low degree of resource utilization, coal gangue is the main source of industrial solid waste in China, and it has a significant impact on the ecological environment and on mine safety [1,2]. At present, China has invested a great deal of labor and material resources to deal with key scientific and technological problems, aiming to accelerate the resource utilization of coal gangue [3,4]. Adequate research work has been carried out in recent years, and certain research results have been achieved in the fields of coal gangue glass-ceramics [5,6], cementitious materials [7,8,9], concrete aggregate [10,11], and filling materials [12,13], among others. However, although there is a large output of coal gangue, its complex composition, low mechanical strength, and limited economic value have led to low utilization and high stockpiles. Therefore, its large-scale processing and utilization require further and continuous exploration.

One of the main ways to utilize coal gangue is as a road stabilization material, and this has attracted the attention of many scholars. Xuchao et al. [14] highlighted the broad prospects for using coal gangue as roadbed filler. Through experiments and application research, Yu et al. [15] discovered that lime, fly ash, and coal gangue can be used in the production of highway pavement base materials. Shangjun [16] proposed that the coal gangue-stabilized base has higher compressive strength, higher tensile strength, better water stability, and frost resistance than the ordinary crushed stone-stabilized base, which can save up to 39.2% of the cost. However, the current materials used to cement the gangue road stabilization layer are mainly cement-based cementing systems. The strength of the stabilized materials takes a long time to develop, and there are many durability problems [17]. Moreover, the production of cement is a process involving high pollution and energy consumption, which is contrary to the theme of green building materials. The identification of inorganic industrial by-products that can replace Portland cement, and the use of industrial by-products [18,19,20,21] to reduce pollution and the environmental burden, have become research hotspots in environmental protection. The alkali-activated gel material is a mixture of aluminosilicate and alkali metal hydroxide or alkali metal silicate solution [22,23], which is an inorganic polymer. At present, the most widely used aluminosilicate materials in alkali-activated gel materials are fly ash and metakaolin. Studies have shown that alkali-reactive material concrete has excellent strength, corrosion resistance, fire resistance, and durability, and it can replace Portland cement as a concrete adhesive [24,25,26]. Therefore, alkali excitation technology is expected to become an important part of sustainable by-products. Alkali-activated materials became a subject of research interest because of their significantly lower environmental impact and superior performance when compared to those of Portland cement. Theoretically, this material can be produced by using any solid precursor containing active calcium, silicate, and aluminate to form C–A–S–H and N–A–S–H gels similar to Portland cement hydration products [27,28]. Wang [29] studied the effect of different silicates on the strength of alkali-activated fly ash and slag. Nur [23] studied the effect of sintering temperature on the microstructure, phase analysis, and electrical conductivity of alkali-activated materials. Guilherme Jorge Brigolini Silva [30] and others have used biomass fly ash and glass powder as precursor materials to produce alkali-activated gel materials and found that, with the increase in NaOH concentration and the decrease in glass powder content, the compressive strength and the flexural strength showed improvements, but there was still larger porosity.

With the strategic goal of carbon neutrality, the use of alkali-activated slag as cementing material for the production of a solid-waste coal gangue road stabilization layer is proposed in this paper, which could make full use of its energy savings, environmental protection, fast hardening, early strength, excellent corrosion resistance, fire resistance, and durability [31,32,33]. By studying the relationship between the coal gangue aggregate rate and the mechanical properties of stable materials under different alkali equivalents, the feasibility of alkali-stimulated slag-cemented coal gangue road stabilization materials is analyzed, and its structural characteristics are explained via microstructure analysis, providing a basis for the development of solid-waste coal gangue road stabilization materials.

## 2. Test

### 2.1. The Raw Material

**Figure 1:**
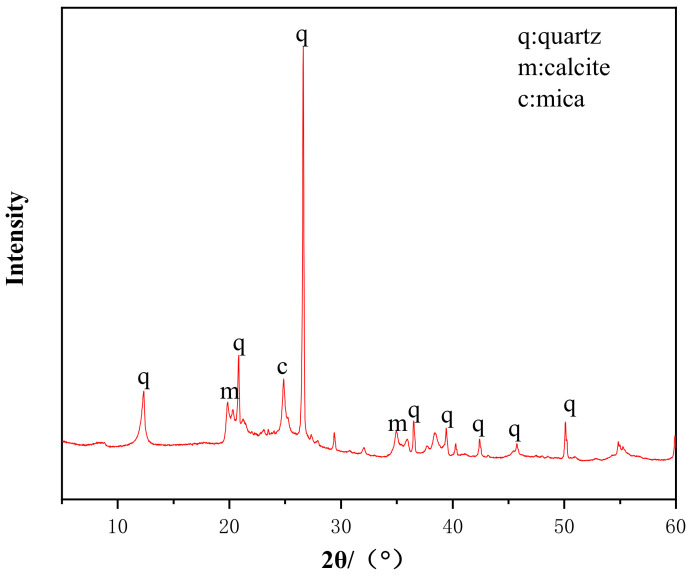
X-ray diffraction patterns of coal gangue.

The chemical composition of coal gangue from Zhangji Mine in Huainan is shown in Table 1 (measured by X-ray fluorescence spectrometry (Thermo Fisher Scientific, Waltham, MA, USA) shown in Figure 1). The slag is S105 grade blast-furnace slag, and its chemical composition is shown in Table 2. Analytical-grade pure tablet NaOH was purchased from Shanghai Jing’an District Sinopharmaceutical Chemical Reagent Co., Ltd, Shanghai, China.

Coal gangue samples were screened according to the corresponding steps in the JTG3430 regulation [34], and about 8 kg of each sample was taken for parallel tests; the experimental data showed that the water absorption rate of the coal gangue was 4.14%.

### 2.2. Material Ratio

In order to clarify the effects of aggregate rate and alkali equivalent (Na_2_O, wt.%) on the mechanical properties and microstructure of the specimens, the specimens with an aggregate rate of 80% and an alkali equivalent rate of 3% were taken as the reference group. The serial numbers of groups with other aggregate rates and alkali equivalents are G70N5, G75N5, G80N5, G85N5, G80N1, G80N3, and G80N7, as shown in Table 3.

The mix ratio of specific samples is listed in Table 3. First, water was taken and NaOH was fully stirred to produce solutions with different alkaline equivalents that were cooled to room temperature. Afterward, the coal gangue and slag were successively added to the mixer. After stirring for 3 min, the alkali solution was slowly added. After stirring for 5 min, the mixture was quickly poured into the mold with a size of 70.7 mm × 70.7 mm × 70.7 mm, and the mold was vibrated on the electric vibrating table for 60 s to be compacted. After curing for 1 day, the mold was removed in the standard curing room (temperature 20 ± 3 °C, relative humidity > 95%) specifically for testing at the ages of 7 and 28 days. Compressive strength tests for the solid-waste coal gangue road stabilization material cube at 7 and 28 days were carried out using a WDT-100 pressure-testing machine (Wugao Electric Technology Co., Ltd, Wuhan, China). The loading rate was 0.1%. One test group contained three test blocks, and the average values of test results were recorded and analyzed.

### 2.3. Test Methods

#### 2.3.1. Microstructure

Specimens that were representative of those used in 7- and 28-day compressive strength tests were soaked in ethanol to stop hydration. After that, they were vacuum-dried and sprayed with gold particles to improve the electrical conductivity. The micromorphology of the samples was observed by scanning electron microscopy (SEM) using a FlexSEM1000 (Hitachi High-Tech Corporation, Tokyo, Japan) with an acceleration voltage of 15 kV.

#### 2.3.2. Porosity Test

The porosity, true density, and apparent density of the test blocks were tested according to ASTM C20 [35]. The three test blocks were taken as a group in the same way, and the measurement results were averaged. The specific method was to place the test block in an oven, dry it to constant weight at 105 °C, and measure its dry weight. After that, the test block was boiled for 2 h and soaked in water for 12 h, and the hanging weight was measured by a thin wire hanging in the water. At the same time, the test block was dried, and its saturated weight was measured. The porosity, true density, and apparent density were converted according to Equations (1)–(3):

(1) $$n=\frac{Q_{3}-Q_{1}}{Q_{3}-Q_{2}}\times 100\%$$

(2) $$\rho_{1}=\frac{Q_{1}}{Q_{1}-Q_{2}}$$

(3) $$\rho_{b}=\frac{Q_{1}}{Q_{3}-Q_{2}}$$

where *n* is the porosity; *Q*_1_ is the dry weight; *Q*_2_ is the hanging weight; *Q*_3_ is the saturated weight; *ρ*_1_ is the true density; and *ρ*_b_ is the apparent density.

## 3. Results and Discussion

### 3.1. Mechanical Properties of Whole-Solid-Waste Coal Gangue Road Stabilization Materials

**Figure 2:**
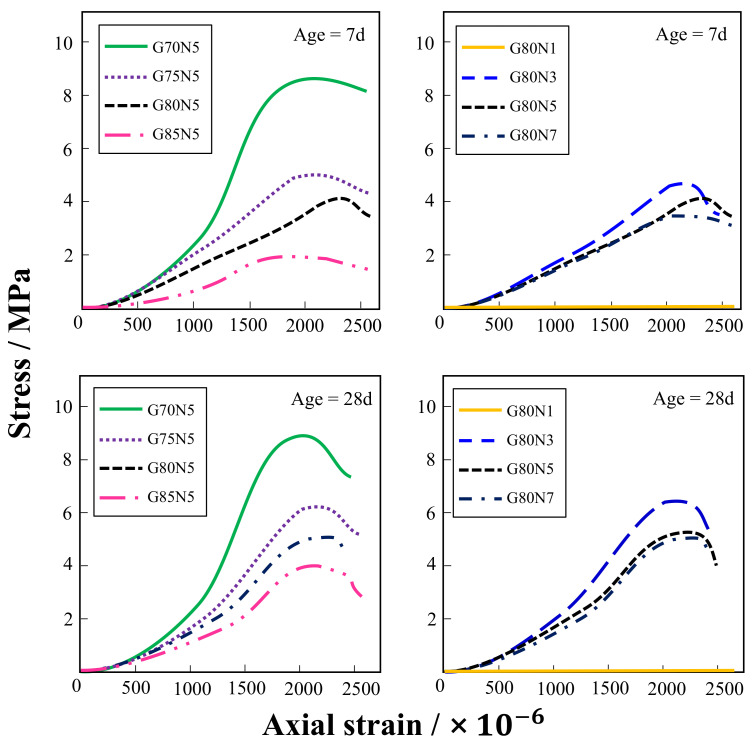
Stress–strain curve of coal gangue road stabilization material at different ages.

The stress–strain curves of whole-solid-waste coal gangue road stabilization materials of different ages are shown in Figure 2. Under the conditions of different aggregate ratios and alkali equivalents, the characteristics of the stress–strain curves are essentially the same: the stress of the sample increases slowly with the increase in strain and finally reaches the peak value. Under the condition of a different aggregate ratio, G70N5 has the highest peak value, and the slope of its rising curve is also the largest, indicating that the specimen has the best compressive strength at a condition of 70% aggregate ratio. In the case of different alkali equivalents, the peak value of G80N3 is the highest, and the slope of the ascending curve is the largest, indicating that the specimen has the best compressive strength in the case of a 3% alkali equivalent. Since the test was stopped at peak stress, no information about the residual stress after the failure of the test block was obtained. However, after peak stress was reached, the curve presented an arc shape without a sudden downward trend, indicating that the test piece still showed a certain plastic deformation ability in the post-peak stage. This is because the concrete will form a “strong and weak” stress structure in the gangue concrete system, due to the weak mechanical properties of gangue. Therefore, in the process of a gradual stress increase, the gangue will be damaged before the cementing mortar to a certain extent, which can buffer the strain energy and form a certain plastic deformation ability. This indicates that the solid-waste coal gangue road stabilization material has a certain capacity for shaping and energy consumption, which is of positive significance when bearing the vibration stress caused by vehicle tires.

**Figure 3:**
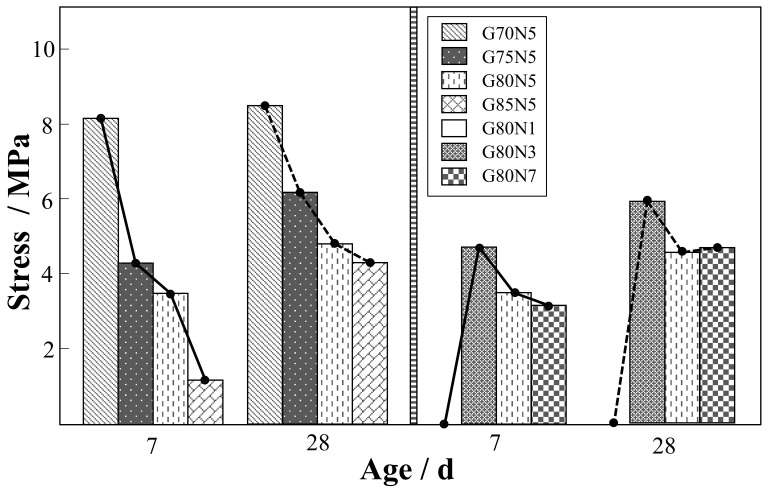
Influence of different alkali contents and aggregate ratios on the compressive strength of the tested block.

Figure 3 shows the influence of different alkali equivalents and aggregate ratios on the compressive strength of the specimens. It can be seen that the aggregate ratio and alkali equivalent have a great influence on the compressive strength of specimens at different ages. At an age of 7 days, the compressive strength of the specimens shows a decreasing trend with the increase in the aggregate ratio. The compressive strength of G75N5 decreases by a maximum of 48.41%. At an age of 28 days, the compressive strength of the specimens also decreases with the increase in the aggregate ratio, but the overall decreasing trend gradually shows a linear relationship. The above two curves indicate that the hydration products generated cannot fully cement coal gangue with an increase in the aggregate ratio, leading to a decrease in strength.

The compressive strength of the specimens, as assessed at days 7 and 28, first increases and then decreases with the increase in the alkali equivalent. The strength of G80N1 is 0, indicating that the gangue cannot be cemented with a low-alkali equivalent, and little gel formation is observed. When the alkali equivalent exceeds 3%, the strength of the specimens begins to decline, indicating that the high alkali equivalent would not be conducive to the improvement in strength, and the best alkali equivalent in terms of strength is 3%.

With the analysis of compressive strength, it is determined that the solid-waste coal gangue road stabilization material has superior strength under the conditions of 70% gangue aggregate ratio and 3% alkali equivalent.

### 3.2. Porosity Test

Porosity and density are two important test indexes to determine the performance of a specimen. Porosity refers to the percentage of the pore volume in the bulk material and the total volume of the material in the natural state, which directly reflects the compactness of the material. The high porosity of a material indicates its small degree of compactness. The results for the porosity and density of the test blocks are shown in Table 4.

It can be noted that the porosity and density values of the specimens are in accordance with the increase in aggregate rate. The unit of the coal gangue mixed with sodium hydroxide decreases with the increase in aggregate rate, leading to lower coal gangue hydration efficiency and a lower quantity of generated gel. Moreover, the larger amount of porosity also decreases the compressive strength. This is consistent with the conclusions from the compressive strength test, as previously discussed.

In the case of different alkaline equivalents, the porosity slightly increases with the increase in the alkaline equivalent, and the porosity is minimal when the alkali equivalent is 3%. This indicates that an excessively high alkaline equivalent does not effectively reduce the porosity [3], which is also one of the reasons why the strength of the specimen decreases when the alkaline equivalent exceeds 3%.

### 3.3. Microstructure Analysis

**Figure 4:**
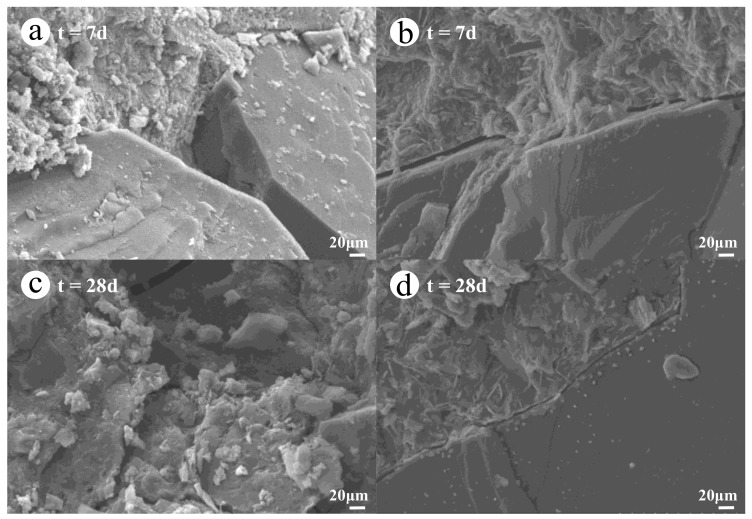
SEM of the G70N5 blocks: (**a**,**b**) t = 7 days; (**c**,**d**) t = 28 days.

In order to analyze the microstructure of whole-solid-waste coal gangue road stabilization materials, typical test blocks of G70N5 at days 7 and 28 were taken as the study samples, as shown in Figure 4.

It can be seen that C–(A)–S–H gel and a small amount of ettringite are generated by the reaction between the activator and the slag. However, due to the short hydration time, the interface between the coal gangue and alkali excitation material is relatively loose, and the porosity is large. As the hydration time increases, as shown in Figure 4c,d, a large number of hydration products are generated in the specimens which are closely attached to the surface of and interwoven with the coal gangue to fill the pores, thereby causing the slurry and coal gangue to be cemented in close proximity. Due to the small amount of cementing materials, there are cracks and pores at the interface between the coal gangue and slurry. However, with the increase in hydration time, the pores are constantly filled, which promotes the formation of a dense microstructure.

## 4. Conclusions

This paper studies the preparation of solid-waste coal gangue road stabilization materials using alkali-activated slag as the cementing material, and the relationship between different aggregate rates and alkali equivalents, and the mechanical properties and microstructure of the stabilized materials are analyzed. The following conclusions are drawn:

- As the alkali equivalent increases from 1% to 7%, the unconfined compressive strength of the alkali-activated gangue road stabilization materials at first increases and then decreases, and 3% is the optimal group (4.69 MPa on day 7 and 5.88 MPa on day 28);
- When the gangue aggregate rate increases from 70% to 85%, the NaOH of the unit gangue mixture decreases with the increase in the gangue aggregate rate, the hydration reaction rate slows down, and the cementite content decreases. Hence, the higher the porosity of the mixture, the lower the compressive strength of the mixture;
- It is feasible to use alkali-activated slag as a cementing material to prepare a whole-solid-waste coal gangue road stabilization material that can meet standard requirements. This material has the characteristics of fast hardening, early strength, and, compared with the ordinary crushed stone stabilized base, costs can be saved by 39.2%.

The research results of this paper are applicable to new roads and can provide a theoretical reference and technical basis for the large-scale resource utilization of coal gangue.

## Figures and Tables

**Table 1:** Chemical composition of coal gangue.

Materials	Chemical Composition/% (XRF)							
	SiO_2_	Al_2_O_3_	Fe_2_O_3_	CaO	MgO	K_2_O	Na_2_O	TiO_2_
Coal gangue	56.26	27.48	10.73	1.56	0.48	0.21	0.77	1.51

**Table 2:** Chemical composition of slag.

Materials	Chemical Composition/% (XRF)						
	CaO	SiO_2_	Al_2_O_3_	MgO	Fe_2_O_3_	TiO_2_	K_2_O
Coal gangue	43.70	26.50	18.20	4.90	1.00	1.00	0.80

**Table 3:** Mixing proportions.

Mixture	Gangue	Slag	NaOH	Water
G70N5	70	20.000	1.290	10.000
G75N5	75	16.667	1.075	8.333
G80N5	80	13.333	0.860	6.667
G85N5	85	10.000	0.645	5.000
G80N1	80	13.333	0.172	6.667
G80N3	80	13.333	0.516	6.667
G80N7	80	13.333	1.204	6.667

Note: The unit is g/100 g.

**Table 4:** Physical properties of test blocks.

Mixture	Porosity/%	True Density (g/cm^3^)	Apparent Density (g/cm^3^)
G70N5	6.28	1.70	1.59
G75N5	7.84	2.23	2.09
G80N5	8.10	2.77	2.54
G85N5	9.16	2.35	2.13
G80N3	6.34	2.46	2.24
G80N7	8.88	2.41	2.22

## Data Availability

The data is available within the article and can be requested from the corresponding author.
